# A Novel Sensitive Immunoassay Targeting the 5-Methylthio-d-Xylofuranose–Lipoarabinomannan Epitope Meets the WHO's Performance Target for Tuberculosis Diagnosis

**DOI:** 10.1128/JCM.01338-18

**Published:** 2018-11-27

**Authors:** George B. Sigal, Abraham Pinter, Todd L. Lowary, Masanori Kawasaki, Andra Li, Anu Mathew, Michael Tsionsky, Ruixiang Blake Zheng, Tatiana Plisova, Ke Shen, Kiyonori Katsuragi, Alok Choudhary, William J. Honnen, Payam Nahid, Claudia M. Denkinger, Tobias Broger

**Affiliations:** a FIND, Geneva, Switzerland; b Meso Scale Diagnostics, LLC, Rockville, Maryland, USA; c Public Health Research Institute Center, New Jersey Medical School, Rutgers University, Newark, New Jersey, USA; d Department of Chemistry, Alberta Glycomics Centre, University of Alberta, Edmonton, AB, Canada; e Otsuka Pharmaceutical Co., Ltd., Tokyo, Japan; f Immunoprecise Antibodies Ltd., Victoria, British Columbia, Canada; g Division of Pulmonary and Critical Care Medicine, University of California, San Francisco, San Francisco, California, USA; UNC School of Medicine

**Keywords:** biomarker, diagnostics, immunoassays, lipoarabinomannan, tuberculosis

## Abstract

The only currently commercialized point-of-care assay for tuberculosis (TB) that measures lipoarabinomannan (LAM) in urine (Alere LF-LAM) has insufficient sensitivity. We evaluated the potential of 100 novel monoclonal antibody pairs targeting a variety of LAM epitopes on a sensitive electrochemiluminescence platform to improve the diagnostic accuracy. In the screening, many antibody pairs showed high reactivity to purified LAM but performed poorly at detecting urinary LAM in clinical samples, suggesting differences in antigen structure and immunoreactivity of the different LAM sources. The 12 best antibody pairs from the screening were tested in a retrospective case-control study with urine samples from 75 adults with presumptive TB. The best antibody pair reached femtomolar analytical sensitivity for LAM detection and an overall clinical sensitivity of 93% (confidence interval [CI], 80% to 97%) and specificity of 97% (CI, 85% to 100%). Importantly, in HIV-negative subjects positive for TB by sputum smear microscopy, the test achieved a sensitivity of 80% (CI, 55% to 93%). This compares to an overall sensitivity of 33% (CI, 20% to 48%) of the Alere LF-LAM and a sensitivity of 13% (CI, 4% to 38%) in HIV-negative subjects in the same sample set. The capture antibody targets a unique 5-methylthio-d-xylofuranose (MTX)-dependent epitope in LAM that is specific to the Mycobacterium tuberculosis complex and shows no cross-reactivity with fast-growing mycobacteria or other bacteria. The present study provides evidence that improved assay methods and reagents lead to increased diagnostic accuracy. The results of this work have informed the development of a sensitive and specific novel LAM point-of-care assay with the aim to meet the WHO's performance target for TB diagnosis.

## Abstract (precis)

The only currently commercialized point-of-care assay for tuberculosis (TB) that measures lipoarabinomannan (LAM) in urine (Alere LF-LAM) has insufficient sensitivity. We evaluated the potential of 100 novel monoclonal antibody pairs targeting a variety of LAM epitopes on a sensitive electrochemiluminescence platform to improve the diagnostic accuracy.

## INTRODUCTION

Tuberculosis (TB) is the number one cause of death due to infectious disease. In 2016, 10.4 million people fell ill with TB and 1.7 million died from the disease (1). TB is also the most common overall cause of mortality in people living with human immunodeficiency virus (HIV), with an estimated 374,000 deaths in 2016 (1). Most of the deaths from TB could have been prevented by an early diagnosis. Globally, there was a gap of 4.1 million cases between the estimated incident and the reported TB cases. This gap is due, in part, to an underdiagnosis resulting from the limitations of established TB tests and a lack of accurate, inexpensive, and rapid tests suitable for typical primary care settings in low- and middle-income countries where TB is prevalent (2).

Traditional diagnostic methods are slow (sputum culture) or insensitive (sputum smear microscopy). Modern techniques, such as the Xpert MTB/RIF and Xpert Ultra real-time PCR test, have become more available but require specialized equipment and facilities, are costly, or are otherwise inaccessible to many low- and middle-income countries (3, 4). The limitations of existing sputum-based TB diagnostics are especially acute for people living with HIV, because they are more likely to have extrapulmonary TB or be unable to produce sputum samples.

Mycobacterial antigens in the serum or urine have attracted interest as TB biomarkers that would not require the collection of sputum samples and that could be measured in low-cost immunoassay-based rapid test formats. Lipoarabinomannan (LAM), a mycobacterial cell wall lipopolysaccharide and virulence factor, has been the most studied TB biomarker due to several attractive features: it is bacterially derived, is abundant in the cell wall of Mycobacterium tuberculosis, is heat and protease stable, and has structural epitopes that are unique to M. tuberculosis. There is extensive evidence that LAM is found in the urine of many TB patients (5, 6), and other studies indicate that it may also be found in sputum (7, 8) and blood (9, 10). While several enzyme-linked immunosorbent assay (ELISA) and rapid (lateral flow) tests have been developed, the only LAM test currently on the market for the clinical diagnosis of TB is the Alere Determine TB LAM test (Alere LF-LAM) from Abbott Diagnostics, a lateral flow test for detecting LAM in urine. There is also a new LAM ELISA from Otsuka Pharmaceutical which can quantitatively measure LAM in sputum but not in urine (11).

Despite the strong initial excitement for urinary LAM as a diagnostic target, the adoption of LAM tests has been limited due to their relatively poor clinical sensitivity across the spectrum of incident TB cases. One review of studies evaluating urinary LAM ELISAs found sensitivities ranging from 13% to 51%, with higher values generally being associated with study cohorts having high rates of HIV infection (12). The observation that LAM levels in urine tend to be higher in HIV-positive TB patients than in HIV-negative TB patients, especially in patients with CD4 counts less than 100 cells/μl, has led to renewed interest in urinary LAM assays as a complement to Xpert testing for people living with HIV, given the relatively low sensitivity of Xpert for this population. However, a comprehensive meta-analysis of studies evaluating the Alere LF-LAM test for diagnosing TB in people living with HIV found the test has a limited diagnostic accuracy: 44% pooled sensitivity for TB diagnosis in HIV-positive cases with a 92% pooled specificity in 3,037 patients (13). This analysis led to the WHO recommendation to only use the Alere LF-LAM test to diagnose TB in HIV-positive individuals with CD4 counts ≤100 cells/μl who have TB symptoms (13). Despite its low sensitivity, testing with Alere LF-LAM followed by immediate treatment was shown to significantly reduce mortality in large, pragmatic, randomized, parallel-group multicenter trials in HIV-positive inpatients (14, 15). In addition, the operational advantages of the point-of-care (POC) LF-LAM test has been shown to lead to improved diagnostic yields for HIV-positive TB patients (16).

However, there are major unanswered questions about urinary LAM as a TB biomarker that are critical to understanding whether there is a path to improve its clinical performance and general utility. Most importantly, it is not understood whether the LAM levels in the urine of LF-LAM-negative TB patients—especially HIV-negative patients—are simply too low to be measured by the current test but could be measured by a test with improved detection limits. Some recent reports indicate that improved detection limits could provide improved performance (17). Alternatively, some investigators have proposed that the presence or absence of LAM in urine may be associated with specific pathologies in some patients, such as increases in glomerular filtration due to kidney dysfunction or a dissemination of M. tuberculosis to the kidneys (18–20). Second, the nature and availability of LAM epitopes present in urine are poorly understood. The present study was designed to answer these questions by employing the latest generation of anti-LAM monoclonal antibodies (MAbs), sensitive electrochemiluminescence (ECL) detection, and a high-throughput laboratory immunoassay platform to (i) identify the best combination of antibodies for measuring LAM in urine and (ii) characterize the LAM levels and availability of LAM epitopes in the urine samples of a set of HIV-positive and -negative TB cases and controls.

## MATERIALS AND METHODS

### Antibodies and control materials.

Purified LAM from M. tuberculosis strain Aoyama-B was obtained from Nacalai USA, Inc. (San Diego, USA). Phosphoinositol-capped LAM (PILAM) from Mycobacterium smegmatis and inactive whole-cell lysates of M. tuberculosis and Mycobacterium bovis were obtained from the Biodefense and Emerging Infections Research Resources Repository (BEI, Manassas, VA, USA). For cross-reactivity testing, live whole-cell stocks of a number of different bacterial and fungal strains were obtained from ATCC (Manassas, VA, USA) as vials of lyophilized cells or frozen cell suspensions in glycerol. The lyophilized cells were suspended in 0.5 ml of 2% bovine serum albumin (BSA) in phosphate-buffered saline (PBS). The stock cell suspensions were then serially diluted in the same buffer to create test samples.

To the extent possible, all existing monoclonal and polyclonal anti-LAM antibodies were used, including A194-01 (21), CS-35 (22), FIND 28 (provided by FIND), S4-20, G3, and O-TB (11). The S4-20, G3, and O-TB antibodies (provided by Otsuka Pharmaceutical) possess the same variable regions and have the same epitope specificity in the glycoconjugate mapping assay as MoAb1 (S4-20), MoAb2 (G3), and MoAb3 (O-TB) characterized in Choudhary et al. (21). The same glycoconjugate mapping assay was also used for this study and is described below. A commercial anti-LAM rabbit polyclonal antibody (Viro Poly) was purchased from Virostat, Inc. (Westbrook, ME, USA).

Two additional recombinant rabbit monoclonal antibodies (13H3 and 27D2) and a rabbit polyclonal antibody (Imm Poly) were produced by ImmunoPrecise Antibodies, Ltd. (Victoria, Canada). These monoclonal antibodies were generated by immunization with synthetic LAM-related polyarabinose oligosaccharide fragments coupled to bovine serum albumin (23–25). Briefly, a rabbit was immunized with 65 μg BSA-Ara6 (ID 44), 65 μg BSA-Ara7 (ID 16), and 65 μg BSA-Ara22 (ID 22) and boosted with the same mixture on days 7 and 14 (see Fig. S1 in the supplemental material for a description of the oligosaccharide structures). Peripheral blood mononuclear cells (PBMCs) were collected at day 28 and cultured in 96-well plates. Supernatants were tested by indirect ELISA for binding to immunizing antigens and low cross-reactivity to BSA. The sequences of heavy and light chains of B cells with the desired activity were cloned into separate expression vectors and cotransfected into HEK293 cells. The resulting transfected supernatants were tested by indirect ELISA to identify the antibodies (13H3 and 27D2) with the most reactivity to purified LAM from M. tuberculosis Aoyama-B and heat-killed M. tuberculosis H37Ra. The polyclonal antibody was produced by immunizing a rabbit with a mixture of 250 μg purified LAM from M. tuberculosis Aoyama-B and complete Freund's adjuvant (CFA) and boosting with a mixture of 250 μg purified LAM from M. tuberculosis Aoyama-B, 200 μg heat-killed M. tuberculosis H37Ra, and incomplete Freund's adjuvant (ICFA) at days 28, 47, and 67. Serum collected on day 76 was purified by affinity chromatography on a protein A column.

The oligosaccharide epitopes recognized by each of these MAbs were mapped by measuring the binding of the antibodies to glycan arrays presenting a diverse set of 60 oligosaccharide structures related to mycobacterial cell wall glycans (Fig. S1), according to previously described methods (21, 23). Briefly, oligosaccharide fragments were synthesized as previously described (23–27), conjugated to BSA, and used to generate microarrays. Serial dilutions of antibodies were incubated on the slides for 30 min at 37°C, which were washed and stained for 40 min with fluorescently labeled secondary anti-species antibodies. Fluorescence signals were measured using a GenePix 4000B scanner (Molecular Devices, Sunnyvale, CA, USA), and the intensity of each spot was quantified using Pro Microarray image analysis software, version 6.1.

### LAM immunoassays.

Immunoassays for LAM employing a multiplexed sandwich immunoassay format and electrochemiluminescence (ECL) detection were carried out on commercial instrumentation and multiwell plate consumables from Meso Scale Diagnostics, LLC (MSD) (28). The assays were run in MSD's U-PLEX 96-well plates. On the bottom of each well of the plate, there is a 10-plex array of binding reagents immobilized on an integrated screen-printed carbon ink electrode. The 10 binding reagents each bind to one of a set of 10 proprietary linkers. In U-PLEX assays, different capture reagents are coupled to different linkers. Arrays of the capture reagents in the plates are formed as needed by adding a mixture of the capture antibody-linker conjugates to the well and allowing the linkers to self-assemble on their complementary array elements (or “spots”). Arrays of anti-LAM antibodies were used to compare the performance of multiple capture antibodies in a single multiplexed measurement.

The antibodies were prepared for use in the assays according to the procedures in the U-PLEX package insert. The capture reagents were biotinylated with Sulfo-NHS-LC-Biotin (Thermo Fisher Scientific) and coupled via biotin-streptavidin binding to U-PLEX linkers. The detection antibodies were labeled with the MSD SULFO-TAG ECL label. To prepare the capture antibody arrays, up to 10 antibody-linker conjugates were combined in U-PLEX Stop buffer at a concentration of 2.9 μg/ml per antibody, and 50 μl of this mixture was added to each well of the U-PLEX plates. The plates were incubated for 1 h with shaking to allow the antibody arrays to assemble and then washed. The plates were used immediately or stored at 4°C in a desiccated pouch until needed.

Unless otherwise indicated, the assays were run according to the following protocol using commercial diluents from MSD that include blocking components to prevent nonspecific signals from human anti-mouse antibodies (HAMAs) or other nonspecific antibody binding proteins. Th capture antibody arrays were preformed in a U-PLEX plate as described above. MSD diluent 22 (25 μl) was combined with 25 μl of sample in each well of the U-PLEX plate, and the mixture was incubated with shaking for 1 h at room temperature to bind LAM in the sample to the capture antibody array in the well. After washing the wells to remove the unbound sample, 25 μl of 2-μg/ml SULFO-TAG-labeled detection antibody (in MSD diluent 3 supplemented with casein) was added and incubated for an additional 1 h with shaking to complete the immunoassay sandwich. After washing the wells to remove the unbound detection antibody, the wells were filled with 150 μl of 2× MSD read buffer T, and ECL was measured on an MSD Sector S 600 ECL plate reader. The plate reader applies a voltage to the electrodes in the MSD plates to induce ECL from the bound detection antibodies and uses a cooled charge-coupled-device (CCD) camera to quantitate the light emission from each array spot (28). During the screening for antibody pairs, a number of capture antibody and detection antibody combinations were evaluated. A more detailed assay evaluation was carried out on a specific panel that combined an array of capture antibodies (CS-35, FIND 28, 13H3, 27D2, S4-20, and O-TB) and the A194-01 detection antibody.

To avoid interference from LAM-like contaminants that were observed to leach from the fluidic lines of automated plate washers, a semiautomated plate wash protocol was used: wash solution was added to the wells manually with a multichannel electronic pipette, and the wash solution was aspirated out of the plate using an automated 96-head plate washer.

To calculate LAM concentrations, an eight-point calibration curve with purified M. tuberculosis LAM (diluted in phosphate-buffered saline plus 2% bovine serum albumin) was run in duplicates in each assay plate. The relationship of ECL signal to LAM concentration was fitted to a four-parameter logistic (4-PL) function. LAM concentrations for test samples were calculated by back-fitting ECL signals to the 4-PL fit.

### Preparation of urine samples.

To inactivate any anti-LAM antibodies that may be present, the urine samples were pretreated prior to analysis by heat treatment at 85°C for 10 min.

### Clinical subjects and samples.

For this retrospective case-control study, a total of 75 urine samples were selected from FIND's biobank. These samples were previously collected from adults presenting at primary care sites in Bangladesh (*n* = 5), Peru (*n* = 19), South Africa (*n* = 15), and Vietnam (*n* = 36) with clinical symptoms of TB who had not yet started TB treatment at the time of sample collection. Approval by local ethics committees and informed patient consent were obtained before enrolling the patients, and no personally identifiable information was available to FIND or to the researchers.

FIND uses standardized protocols for the collection and processing of samples. Briefly, urine and venous blood were collected at first contact with the patient and then processed, aliquoted, and frozen (−80°C) on the same day (typically within 4 h). The processing of urine involved centrifugation at 200 × *g* at 4°C for 10 min prior to aliquoting, although there was some variation in the processing protocols for samples collected under different studies. WHO prequalified *in vitro* diagnostics were used for HIV serological testing and CD4 counting. For use in patient classification, sputum samples (typically two in the first 24 h) were also collected from all participants, decontaminated, and tested in up to six independent liquid cultures (MGIT; BD, Franklin Lakes, NJ, USA) and solid cultures (Lowenstein-Jensen medium). The presence of the M. tuberculosis complex in cultures was confirmed by Ziehl-Neelsen staining or auramine O fluorescence microscopy to identify acid-fast bacilli, MPT64 antigen detection using rapid speciation assays (such as the Capilia TB test; TAUNS, Japan), or molecular methods. The urine samples used in this study were also tested using the Alere LF-LAM test run according to the manufacturer's instructions. The strip was read independently by two different technicians who compared the test line intensity with the reference card provided by the manufacturer and graded the results. In case of discordant results, a third technician would interpret the assay to come up with the final result. For documentation, all strips were scanned.

Subjects were classified using a composite reference standard on the basis of clinical and laboratory findings as described elsewhere (29). TB-positive individuals were patients with at least one positive culture. All TB-positive patients had positive microscopy results. Participants who were smear negative and culture negative on ≥4 cultures from all sputum samples and who exhibited symptom resolution in the absence of tuberculosis treatment and negative sputum culture results at a 2-month follow-up visit were classified as TB negative. The subjects were further classified as HIV positive or HIV negative on the basis of HIV rapid tests.

## RESULTS

### Antibody generation and selection.

**FIG 1:**
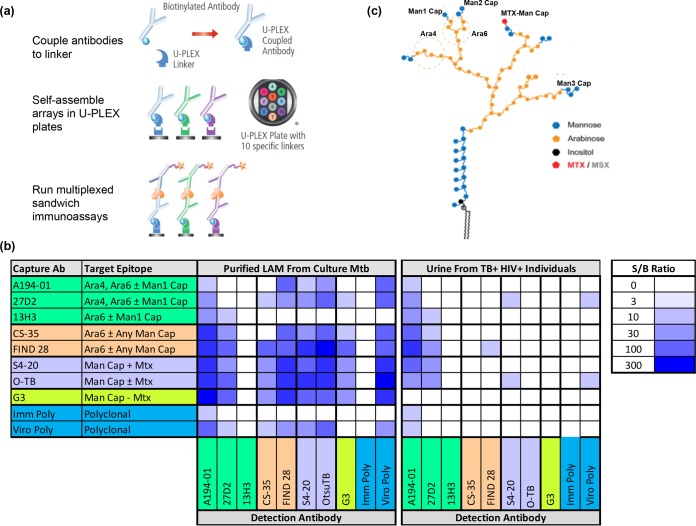
Results of antibody screen to identify antibody pairs for detecting LAM. (a) Schematic of the U-PLEX format used for immunoassay measurements. The U-PLEX plates have an array of binding reagents specific for 10 different U-PLEX “linkers.” Biotin-labeled antibodies are coupled to the linkers and then self-assembled to specific locations on the U-PLEX array. These arrays can then be used to carry out multiplexed sandwich immunoassays using detection antibodies carrying ECL labels. (b) Heat maps that show the ability of each pairwise combination of capture (rows) and detection (columns) antibodies to detect 10 ng/ml of purified LAM from cultured M. tuberculosis (Mtb; left) and a 1:50 dilutions of urine from TB-positive HIV-positive individuals (right). The heat maps display the signal-to-blank (S/B) ratio. The data in the urinary LAM heat map represent the maximum values for urine samples from two individuals. The antibody names are color coded on the basis of the LAM epitopes they target, as determined by binding to glycan arrays, and the epitopes are listed next to the names of the capture antibodies (see Fig. S2 in the supplemental material for details of the epitope mapping results). (c) Schematic of LAM illustrating the different epitopes listed in the heat map. (Adapted from reference 47 with permission of the publisher; kindly provided by Bruce Turnbull.) MTX, 5-methylthio-xylofuranose; MSX, 5-methylsulfoxy-xylofuranose.

To identify potentially useful antibody pairs for use in sandwich immunoassays, we screened a library of anti-LAM MAbs. Each possible pair of capture and detection antibodies (100 pairs in total) was assessed for its ability to detect purified LAM from cultured M. tuberculosis and urinary LAM in urine sample from two TB-positive HIV-positive human subjects with low CD4 blood counts. A multiplexing approach using ECL-based instrumentation and consumables from Meso Scale Diagnostics was employed. This assay platform provided sensitive detection and enabled the pairing of a detection antibody with up to 10 different capture antibodies to be evaluated in parallel in a single well (Fig. 1a). Figure 1b provides heat maps displaying the signal-to-blank ratio (S/B) achieved with each antibody pair and groups and color-codes antibodies based on their specificity for different LAM epitopes as defined in Fig. 1c. Specificities were characterized using glycan arrays in new (see Fig. S1 and S2 in the supplemental material) and prior (21, 23) studies. Many antibody pairs showed high reactivity to purified LAM but were relatively poor at detecting urinary LAM. Only two antibodies (A194-01 and 27D2) were useful as detection antibodies for detecting both purified bacterial LAM and urinary LAM. A194-01 was the more sensitive of the two, giving 2- to 5-fold higher signals in patient urine. Both of these antibodies possessed high affinities for linear tetra-arabinoside (Ara4) and branched hexa-arabinoside (Ara6) structures in the arabinan domain of LAM, and A194-01 also targeted a subset of mannose (Man)-capped structures. The specificity of 27D2 toward Ara4/Ara6 confirmed the utility of synthetic LAM glycans fragments coupled to BSA as immunogens for the development of antibodies with specificity for defined LAM epitopes. Surprisingly, the antibodies requiring the presence of Man-capped arabinose structures tended to provide high signals when used as a detection antibody for measuring purified LAM from cultured M. tuberculosis but not when measuring urinary LAM. An example is the G3 antibody which, on the basis of the glycan array results, recognizes di- and trimannose-capped Ara4 and Ara6 structures and, consistent with previous reports (21), also reacts strongly with several arabinose-free pentamannose structures.

Differences in relative reactivity to LAM from culture and urine were also observed for capture antibodies (Fig. 1b). When combined with A194-01 as the detection antibody, nearly all capture antibodies in our library provided high signals for purified LAM from cultured M. tuberculosis. This includes antibodies targeting branched Ara4/Ara6 (CS-35) and Ara6 (FIND 28) structures, Man2 or Man3 caps (G3 and O-TB), and 5-methylthio-d-xylofuranose (MTX)-Man caps (S4-20), where MTX-Man refers to Man2 or Man3 caps further modified with an MTX residue. In contrast, when measuring the urinary LAM samples, the capture antibodies targeting the more general Ara4/Ara6 motif (CS-35 and FIND 28) tended to give higher signals than the antibodies such as S4-20 and O-TB that target MTX-Man, a structure that is reported to be specific to M. tuberculosis among other mycobacteria (30–32). In agreement with its behavior as a detection antibody, when G3 (which primarily targets Man2 and Man3 caps) was used as a capture antibody, it also provided low signals for urinary LAM. The performance differences between the different categories of capture antibodies were less pronounced than those for the detection antibodies. To enable us to efficiently compare the clinical potential of these binding reagents and to get an increased understanding of the abundance of LAM structures in urine, we evaluated 12 MAb pairs using a multiplexed panel of six capture antibodies covering a range of epitope specificities (CS-35, FIND 28, 13H3, 27D2, S4-20, and O-TB) combined with the two most sensitive detection antibodies (A194-01 and 27D2).

### Analytical assay performance.

Figure S3 in the supplemental material shows the calibration curves of purified bacterial LAM for each of the selected antibodies when combined with A194-01 as the detection antibody. The intraplate coefficients of variation (CVs) for the blank (no LAM) sample were ≤15% for all six capture antibodies (see Table S1). On the basis of these results, we defined the lowest detectable signal as 37.5% above the blank (S/B = 1.375), providing a threshold signal that was at least 2.5 standard deviations above the blank signal for all assays. CVs for the blank signals were dominated by the electronic noise of the system (±30 ECL intensity units as reported by the ECL plate reader); as the signals increased above the blank signals, the CVs decreased considerably. On average, the CVs for LAM levels above this threshold were between 3% and 4% for all 12 pairs. Table S1 and Fig. S3 also provide the limits of detection (LODs) based on the signal threshold for each assay. Due to the higher signal-to-background ratios provided by the FIND 28 and S4-20 capture antibodies, these antibodies provided more sensitive detection of purified LAM calibrators and LODs of 6 and 11 pg/ml, respectively. Assuming an average molecular mass of 17 kDa for LAM, this corresponds to sensitivities in the femtomolar range (350 and 650 fM, respectively). When used as a detection antibody, 27D2 provided results that were highly correlated to results obtained using A194-01 but tended to provide lower signals and higher detection limits. Because of the high correlation and similar epitope specificities of the two antibodies, we focused the subsequent analyses on results obtained with the more sensitive A194-01 detection antibody.

### Sample preparation.

Prior to testing in the assay, urine samples were heat treated at 85°C for 10 min to inactivate any anti-LAM antibodies that may be present in the samples. The testing of a small set of urine samples from TB patients provided no evidence that interfering antibodies were present in these samples, as signals were generally unchanged or only slightly increased by the heat treatment (see Fig. S4). Thus, heat treatment appeared not to be necessary. However, because the heat treatment did not have a negative impact on LAM detection and as we only ruled out the possibility of interference from anti-LAM antibodies in a subset of urine samples, we decided to include this step in testing of all samples. To assess the effect of the urine sample matrix on LAM quantitation, we carried out spike recovery and dilution linearity experiments using urine samples from TB-positive and TB-negative subjects. The average recoveries of LAM spiked into urine samples and LAM measured in diluted urine samples were within 80% to 120% of the expected values for all the selected antibody pairs, with the exception of pairs using the 27D2 capture antibody, which underquantitated the LAM spiked into urine (see Table S2).

### Cross-reactivity for other bacteria.

We tested the LAM assays for cross-reactivity against a panel of 10 different mycobacterium species and 20 different nonmycobacterial microorganisms that could potentially be present in urine samples. Table 1 provides the signal-to-blank ratios measured with each capture antibody when paired with the A194-01 detection antibody. At the highest concentration tested (1:100 dilution of the stock ATCC or BEI materials), only four of the nonmycobacterial species (Nocardia asteroids, Gordonia bronchialis [Tsukamura], Rhodococcus sp., and Tsukamurella paurometabola) provided S/B values greater than the assay threshold of 1.375 for at least one capture antibody.

**TABLE 1:** Analysis of LAM assay cross-reactivity for a set of microorganisms

Species^*a*^	S/B for capture antibody^*b*^					
	CS-35	FIND 28	13H3	27D2	S4-20	O-TB
1:1,000 dilution of mycobacterium species						
M. tuberculosis, H37Rv^*c*^	1,202	14,427	1,342	439	1,977	99
M. bovis^*c*^	551	9,946	778	321	3,324	136
M. fortuitum	668	12,871	556	372	ND^*d*^	691
M. smegmatis	6,717	30,447	4,033	669	ND	1,791
M. abscessus	71	1,255	37	23	ND	109
M. chelonae	19	244	9	5.2	ND	28
M. gordonae^*c*^	11	239	11	4	25	8
M. intracellulare^*c*^	4	66	3	3	129	18
M. avium^*c*^	2	9	ND	ND	1.4	3
M. kansasii^*c*^	5.7	74	4	2	10	7
1:100 dilution of nonmycobacteria^*e*^						
Gordonia bronchialis	5	6	2	9	ND	59
Nocardia asteroides	3	ND	2	25	ND	62
Rhodococcus sp.	16	ND	14	170	ND	359
Tsukamurella paurometabola	4	219	26	57	ND	42
1:100 dilution of other organisms^*f*^	ND	ND	ND	ND	ND	ND

a Dilutions were from stock preparations obtained from ATCC or BEI. All the tested preparations were whole live cells except for M. tuberculosis and M. bovis (killed whole-cell lysates) and M. smegmatis (PILAM purified from cell lysates).

b S/B, signal-to-blank ratio; each paired with A194-01 as the detection antibody. Data are only shown for organisms that gave S/B ratios greater than the assay threshold (1.375) for at least one capture antibody at the listed dilution.

c Slow-growing mycobacterium.

d ND, not detectable (S/B of <1.375 or the signal was too low relative to the signals on the other spots (<0.2%) to accurately measure cross-reactivity).

e S/B of >1.375 for at least one assay.

f Organisms with undetectable cross-reactivity (S/B of ≤1.375 at 1:100 dilution) for all capture antibodies: Candida albicans, Corynebacterium urealyticum, Escherichia coli, Klebsiella pneumoniae, Streptococcus agalactiae, Staphylococcus saprophyticus, Pseudomonas aeruginosa, Staphylococcus aureus, Proteus mirabilis, Proteus vulgaris, Neisseria gonorrhoeae, Haemophilus influenzae, Enterococcus faecalis, Enterobacter aerogenes, and Chlamydia trachomatis.

The strengths of the cross-reactivity for these four species varied considerably across the different capture antibodies. 27D2 and O-TB showed the strongest cross-reactivities for all four species. CS-35, FIND 28, and 13H3 also cross-reacted with the four species but had signals that were one to two orders of magnitude lower. S4-20 provided the best discrimination and did not exhibit measurable cross-reactivity against any of the nonmycobacterial species at the tested concentrations.

All capture antibodies provided strong signals for the TB-causing mycobacterial species M. tuberculosis and M. bovis; the testing of 1:1,000 dilutions of these bacterial preparations gave signals that were above the linear range for the assays. Large differences, however, were observed in the cross-reactivity of the different capture antibodies for other mycobacterial species tested at this dilution. All the capture antibodies except S4-20 provided very high cross-reactivity for the fast-growing Mycobacterium fortuitum and M. smegmatis species and provided signals above the linear range for the 1:1,000 dilution. In contrast, when S4-20 was used as the capture antibody, the cross-reactivity for these two species was at least three orders of magnitude lower than for the other capture antibodies and below the limit of what could be accurately measured in the multiplexed format. With the exception of Mycobacterium intracellulare, S4-20 also tended to have lower cross-reactivity for the other slow-growing mycobacterium species.

Non-TB-causing mycobacteria are known to contaminate water systems and may form biofilms in tubing (33). The use of more specific antibodies such as S4-20 also protects against interference from this environmental source of potential cross-reacting mycobacteria. An example of this is our observation that the exposure to wash fluid dispensed by our automated plate washers resulted in elevated signals, presumably due to a non-TB mycobacterial biofilm present in the washer tubing. This effect was much stronger for the FIND 28 capture than for S4-20. The fluid that contacted this tubing also gave a positive result when tested with the Alere LF-LAM test, suggesting that environmental sources of LAM should generally be considered when developing or evaluating LAM test results. To avoid any possible confounding effects from the washer, we employed a semimanual approach for washing plates during LAM assays: wash fluid was added manually with a multichannel pipettor with disposable tips, and the wash fluid was then removed with the aspiration head of an automated plate washer.

### Clinical assay performance.

The LAM assays were evaluated in a case-control study of 75 subjects, roughly evenly divided between TB-positive HIV-positive (*n* = 25), TB-positive HIV-negative (*n* = 15), TB-negative HIV-positive (*n* = 15), and TB-negative HIV-negative (*n* = 20) subjects. A detailed breakdown of the study population is provided in Table 2. The samples were from FIND's repository of TB clinical samples and were selected to include a range of geographical locations (Asia, Africa, and South America). CD4 counts were available for most of the TB-positive HIV-positive subjects and included subjects above and below the 100 cells/μl threshold used in the WHO algorithm for identifying immunocompromised patients most likely to benefit from the Alere LF-LAM test. In agreement with studies of the clinical performance of the Alere test, the sensitivity of the Alere test for this panel of urine samples was 44% (11/25) for HIV-positive subjects but only 13% (2/15) for HIV-negative subjects.

**TABLE 2:** Characteristics of the study population broken down by TB and HIV status

Category	No. (%) of subjects				
	All Subjects	TB negative		TB positive	
		HIV negative	HIV positive	HIV negative	HIV positive
All subjects	75 (100)	20 (27)	15 (20)	15 (20)	25 (33)
Sex					
Female	21 (28)	6 (8)	3 (4)	5 (7)	7 (9)
Male	49 (65)	9 (12)	12 (16)	10 (13)	18 (24)
NA^*a*^	5 (7)	5 (7)	0 (0)	0 (0)	0 (0)
Age (yr)					
0–20	1 (1)	1 (1)	0 (0)	0 (0)	0 (0)
21–40	45 (60)	5 (7)	9 (12)	12 (16)	19 (25)
41–60	25 (33)	13 (17)	6 (8)	2 (3)	4 (5)
61+	2 (3)	1 (2)	0 (0)	1 (1)	0 (0)
NA	2 (3)	0 (0)	0 (0)	0 (0)	2 (3)
Location					
Bangladesh	5 (7)	5 (7)	0 (0)	0 (0)	0 (0)
Peru	19 (25)	3 (4)	14 (19)	2 (2)	0 (0)
South Africa	15 (20)	2 (3)	0 (0)	5 (7)	8 (10)
Vietnam	36 (48)	10 (13)	1 (1)	8 (11)	17 (23)
CD4 count^*b*^					
≤100 cells/μl	14 (19)	0 (0)	0 (0)	0 (0)	14 (19)
>100 cells/μl	8 (10)	0 (0)	0 (0)	0 (0)	8 (10)
NA	53 (71)	20 (27)	15 (20)	15 (20)	3 (4)
Alere					
Negative	62 (83)	20 (27)	15 (20)	13 (17)	14 (19)
Positive	13 (17)	0 (0)	0 (0)	2 (3)	11 (14)

a NA, not available.

b CD4 cell counts were only available for TB-positive HIV-positive subjects.

**FIG 2:**
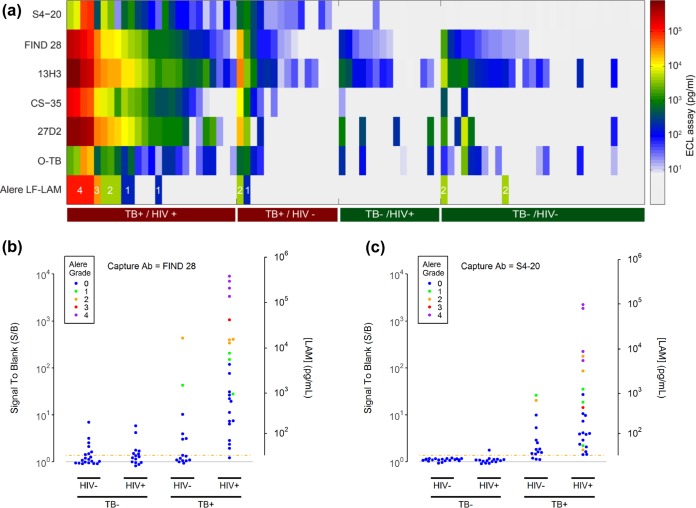
Measured LAM concentrations and assay signals in clinical study samples. (a) Heat map showing the measured LAM concentrations for all tested urine samples (columns) for the six different capture antibodies tested in multiplex format in combination with the A194-01 detection antibody. The samples are grouped by the donors' TB and HIV statuses. The bottom row provides the Alere LF-LAM test grade for each sample for comparison (only samples with positive Alere LF-LAM test results are colored). The results from panel a for the FIND 28 (b) and S4-20 (c) capture antibodies in scatter plot format. The plots show the measured signal-to-blank (S/B) ratios (left axes) and LAM concentrations (right axes) for each urine sample as a function of the TB and HIV status of the donor. The dashed orange lines show the assay threshold (S/B = 1.375). Concentration values are only meaningful for points above the assay threshold. The points are colored by the results of the Alere LF-LAM test for the same samples. Scatter plots for the other 4 capture antibodies can be found in Fig. S5 in the supplemental material.

Figure 2a is a heat map that shows the measured LAM concentrations for the full sample set as a function of TB and HIV status. The heat map compares the concentrations measured with the six capture antibodies, with A194-01 as the detection antibody. All the capture antibodies showed measurable concentrations of LAM in most of the urine samples from HIV-positive TB-positive subjects, but only S4-20, FIND 28, and 13H3 detected LAM in urine from a significant proportion of the HIV-negative TB-positive subjects. Of these three, only S4-20 provided good discrimination of TB-positive and TB-negative subjects. In contrast, FIND 28 and 13H3 detected LAM or LAM-related structures in urine from many of the TB-negative subjects. The differences in performance for the S4-20 and FIND 28 capture antibodies are shown more clearly in the scatter plots in Fig. 2b and c (comparable plots for the other capture antibodies can be found in Fig. S5). Qualitatively, the signals from FIND 28 and S4-20 for samples from TB-positive donors were well separated from the assay threshold. The performance of these two antibodies with TB-negative samples, however, was considerably different. FIND 28 gave a widespread distribution of signals for TB-negative samples, with one sample giving a signal as high as 10 times the signal for a blank sample. In contrast, the signals for TB-negative samples using the more TB-specific S4-20 capture antibody were tightly packed near the blank signal, with the highest signal for a TB-negative sample having an S/B value of approximately 1.8 and all other samples providing signals below the LOD of 11 pg/ml. Color coding according to Alere LF-LAM test results shows that the LAM signals detectable with the Alere test are one to two orders of magnitude above the detection limits for the ECL assays using the FIND 28 or S4-20 capture antibodies, and that there were a large number of samples from TB-positive subjects that were detectable with the ECL assays but not the Alere test.

Table 3 provides the measured sensitivity and specificity of the LAM assays for the test sample set. As an indicator of the separation between the assays signals for the TB-negative and TB-positive groups, the table also provides the area under the curve (AUC) values from the receiver operating characteristic (ROC) curve analysis. Due to the combination of high signals for TB-positive samples (including samples from HIV-negative subjects) and the tight distribution of signals below the LOD for TB-negative samples, the AUC value for the assay using the S4-20 capture antibody (0.98 [0.95 to 1.00]) was significantly better than the AUC value for FIND 28, which gave the next best result (0.84 [0.75 to 0.93]). When examining only the HIV-negative samples, the difference between S4-20 (0.95 [0.87 to 1.00]) and FIND 28 (0.67 [0.48 to 0.85]) was even greater.

**TABLE 3:** Accuracy of LAM assays as evaluated using samples from case-control cohort^*a*^

HIV status	Capture Ab	Sensitivity^*b*^		Specificity^*c*^		AUC (95% CI)^*d*^
		No. correct/total	% (95% CI)^*e*^	No. correct/total	% (95% CI)^*e*^	
All	S4-20	37/40	93 (80–97)	34/35	97 (85–100)	0.98 (0.95–1.00)
	FIND 28	31/40	78 (62–88)	22/35	63 (46–77)	0.84 (0.75–0.93)
	13H3	28/40	70 (55–82)	30/35	86 (71–94)	0.81 (0.71–0.91)
	CS-35	20/40	50 (35–65)	34/35	97 (85–100)	0.76 (0.65–0.88)
	27D2	14/40	35 (22–50)	34/35	97 (85–100)	0.73 (0.62–0.85)
	O-TB	21/40	53 (37–67)	28/35	80 (64–90)	0.73 (0.61–0.84)
	Alere test	13/40	33 (20–48)	35/35	100 (90–100)	0.66 (0.59–0.74)
HIV negative	S4-20	12/15	80 (55–93)	20/20	100 (84–100)	0.95 (0.87–1.00)
	FIND 28	7/15	47 (25–70)	13/20	65 (43–82)	0.67 (0.48–0.85)
	13H3	6/15	40 (20–64)	17/20	85 (64–95)	0.60 (0.40–0.80)
	CS-35	3/15	20 (7–45)	19/20	95 (76–100)	0.48 (0.26–0.70)
	27D2	2/15	13 (4–38)	19/20	95 (76–100)	0.50 (0.30–0.70)
	O-TB	4/15	27 (11–52)	16/20	80 (58–92)	0.54 (0.34–0.74)
	Alere test	2/15	13 (4–38)	20/20	100 (84–100)	0.57 (0.48–0.66)
HIV positive	S4-20	25/25	100 (87–100)	14/15	93 (70–100)	0.99 (0.97–1.00)
	FIND 28	24/25	96 (80–100)	9/15	60 (36–80)	0.96 (0.91–1.00)
	13H3	22/25	88 (70–96)	13/15	87 (62–96)	0.96 (0.90–1.00)
	CS-35	17/25	68 (48–83)	15/15	100 (80–100)	0.89 (0.78–1.00)
	27D2	12/25	48 (30–67)	15/15	100 (80–100)	0.89 (0.80–0.99)
	O-TB	17/25	68 (48–83)	12/15	80 (55–93)	0.88 (0.76–0.99)
	Alere test	11/25	44 (27–63)	15/15	100 (80–100)	0.72 (0.62–0.82)

a Results are for assays using A194-01 as a detection antibody. Results from Alere test are included for comparison.

b Correctly classified TB-positive samples/total number of TB-positive samples.

c Correctly classified TB-negative samples/total number of TB-negative samples.

d AUC values from ROC analysis including confidence intervals (CIs) as determined by bootstrapping.

e Confidence intervals calculated using Wilson's method.

The AUC differences were reflected in the higher observed accuracy of the assay using S4-20 (overall sensitivity, 93% [80% to 97%; 37/40]; specificity, 97% [85% to 100%; 34/35] at a cutoff of 11 pg/ml), relative to FIND 28 (overall sensitivity, 78% [62% to 88%; 31/40]; specificity, 63% [46% to 77%; 22/35] at a cutoff of 6 pg/ml). The assay using the S4-20 capture antibody was approximately three times more sensitive than the Alere LF-LAM assay (overall sensitivity, 33% [20% to 48%; 13/40]; specificity, 100% [90% to 100%; 35/35]) while maintaining high specificity. Assays using both the S4-20 and FIND 28 capture antibodies were perfect or near perfect in identifying TB-positive HIV-positive samples (S4-20 sensitivity,100% [87% to 100%; 25/25]; FIND 28 sensitivity, 96% [80% to 100%; 24/25]); the differences in overall sensitivity were primarily due to the excellent performance of the assay using S4-20 in identifying TB-positive HIV-negative samples (S4-20 sensitivity, 80% [55% to 93%; 12/15]; FIND 28 sensitivity, 47% [25% to 70%; 7/15]).

**FIG 3:**
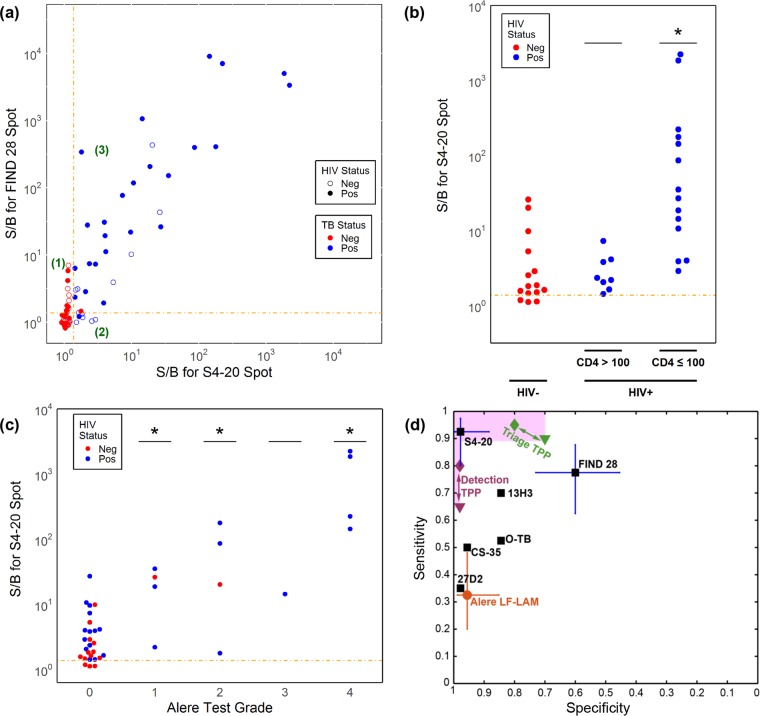
Analysis of LAM assay performance. (a) Correlation of assay signals measured with FIND 28 and S4-20 capture antibodies paired with A194-01 as a detection antibody. Region 1, points that are false positives for FIND 28 but not S4-20; region 2, points that are low true positives for S4-20 but are undetectable with FIND 28; region 3, points with signals above the cutoffs for both FIND 28 and S4-20. Assays signals for TB-positive subjects broken down by HIV status and CD4 count (in cells per μl) (b) and Alere LF-LAM test grade (c). *, *P* < 0.05 versus the left-most group by Mann-Whitney test. (d) The observed clinical sensitivity and specificity (with 95% confidence intervals) for each candidate capture antibody when paired with the A194-01 detection antibody. The plot also shows the minimal (triangle) and optimal (diamond) target sensitivity and specificity requirements set by the WHO in its target product profile (TPP) requirements document for POC TB tests (42) used for two different use case scenarios: (i) definitive detection/diagnosis of TB (purple symbols) or (ii) triage to identify patients who should undergo further confirmatory testing for TB (green symbols). The marker representing the performance of an assay would ideally be above and to the left of the marker representing the requirement for a use case (the area of interest is highlighted).

Figure 3a shows the correlation of the LAM assay signals obtained using the S4-20 and FIND 28 capture antibodies. Qualitatively, the signals generally correlate, although there are some differences. While the signals obtained using the two capture antibodies for purified bacterial LAM are nearly identical (Fig. S3), the FIND 28 assay gave higher signals for urine with high LAM concentrations than the S4-20 assays (median ratio, ∼5). This ratio is also variable, and there are several samples that showed markedly lower LAM levels with the S4-20 assay (Fig. 3a, points in region 3). At low LAM levels, the S4-20 assay performed better with several TB-positive samples giving low but detectable LAM signals in the S4-20 assay, while being undetectable in the FIND 28 assay (Fig. 3a, region 2). In addition, there were a number of TB-negative samples that gave high false-positive signals with FIND 28 but were undetectable with S4-20 (Fig. 3a, region 1).

The associations of assay signals (using the S4-20 antibody) with CD4 counts are shown in Fig. 3b and with Alere LF-LAM test results in Fig. 3c. The increased LAM levels correlated with immunosuppression. HIV-positive subjects that were strongly immunosuppressed (CD4, <100 cells/μl) had significantly higher levels of LAM than HIV-negative subjects. There was no significant difference between HIV-positive subjects with high CD4 counts (>100 cells/μl) and immunocompetent HIV-negative subjects (expected to have CD4 counts >200 cells/μl). Confirming the qualitative findings from Fig. 2b and c, Fig. 3c shows that high Alere LF-LAM grade is associated with very high ECL assay signals. The figure also highlights the significant number of TB-positive subjects that had low but detectable signals by the ECL assay but were undetected with the Alere test.

## DISCUSSION

We have developed a novel ECL assay using an optimized pairing of monoclonal antibodies (capture S4-20/detector A194-01) that provides an almost 3-fold higher sensitivity and statistically indistinguishable specificity for TB case detection compared with the Alere LF-LAM assay for a set of 75 urine samples from well-characterized patients that presented with TB-like symptoms. All the HIV-positive TB-positive subjects and a significant fraction of HIV-negative TB-positive subjects had detectable LAM concentrations above the assay detection limit of 11 pg/ml (650 fM). The femtomolar detection limit of the ECL assay was 25- to 50-fold below the cutoff of the Alere LF-LAM test, which lies in the range of 250 to 500 pg/ml (34, 35). The results suggest that improvements in analytical sensitivity for the detection of LAM can directly lead to improvements in clinical sensitivity for diagnosing TB. Our findings are consistent with other reports that suggested that low concentrations of LAM and other M. tuberculosis antigens can be detected in the urine samples of immunocompetent TB patients through the use of improved assay methods. For example, Hamasur and colleagues used magnetic beads for sample concentration prior to LAM detection to achieve an LOD of 50 pg/ml and reported high sensitivity and specificity in a small case-control study with HIV-negative subjects (36). In more recent studies, Paris et al. (17) developed a sample preparation device that preserves and concentrates antigens and managed to quantify LAM down to 14 pg/ml, leading to 95% sensitivity but relatively low 80% specificity in a case-control study with 48 HIV-negative TB-positive subjects and 53 negative controls. Also, in a limited study using the CS-35/A194-01 combination and a standard enhanced chemiluminescence protocol, Choudhary et al. were able to detect positive signals for LAM in 7 of 10 TB-positive HIV-negative urine samples (21), and another study using this MAb pair and a protease pretreatment and preconcentration step reported high accuracy (46). In contrast to these studies, our assay does not require a preconcentration step and the assay format is translatable to an easy-to-use POC test. A highly sensitive yet simple lateral flow immunoassays has already been developed that detects picogram per milliliter concentrations of histidine-rich protein II (HRP2) for malaria diagnosis, and a similar approach could be taken (37). All studies, including our own, are small case-control studies and further validation in larger cohorts is required.

The key driver for the increased diagnostic sensitivity with nearly perfect specificity for the ECL assay was the identification of a pair of well-defined monoclonal antibodies with binding specificities to distinct LAM epitopes that are present in the urine samples of TB patients. In a screen of each possible pairwise combination of a set of anti-LAM antibodies from different sources, we found many pairs that were able to detect purified LAM from M. tuberculosis culture, but only a small subset showed good sensitivity for detecting LAM or LAM-related structures in patient urine. The choice of detection antibody appeared to be especially important for the sensitive detection of LAM in urine, and we identified two antibodies (A194-01 and, to a lesser extent, 27D2) that provided substantially better performance as detection antibodies than the other candidates. The enhanced activity of A194-01, an antibody that was isolated from a TB-infected human donor, may be associated with its relatively unique ability to target both uncapped and capped Ara4 and Ara6 motifs (21).

As most of the candidate antibodies worked reasonably well as capture antibodies for detecting bacterial LAM spiked into urine, the selection of an optimal capture antibody was primarily driven by antibody specificity for the urinary form of this antigen. Antibodies targeting the linear Ara6 motif (FIND 28), or both the linear Ara4 and branched Ara6 motifs (A194-01, CS-35, and 27D2), when paired with A194-01, were all able to detect LAM in at least some of the urine samples. Of this set of arabinan-specific capture antibodies, FIND 28 provided the lowest detection limit and tended to have the highest signals for urine from TB-positive subjects but showed poor specificity (63%), with some TB-negative samples giving signals as high as 10-fold above the blank signal. While we were able to develop a LAM assay using the FIND 28/A194-01 pair that provided excellent analytical sensitivity, our results suggest that the performance of urinary LAM assays using only antibodies targeting non-TB specific arabinan epitopes may ultimately be limited by cross-reactivity with urinary LAM from other sources, such as non-TB mycobacteria (NTMs) or related organisms of the Actinomycetales order. In particular, the Ara6 structures are not unique to M. tuberculosis LAM, and our cross-reactivity studies confirmed that the FIND 28/A194-01 pair cross-reacts with the nonmycobacterial actinomycetes Nocardia, Gordonia, Rhodococcus, and Tsukamurella, which are all known to produce LAM with Ara6 structures (38, 39). It is likely that CS-35 and the polyclonal antibodies used in the Alere LF-LAM test and previous commercial ELISAs have similar limitations in specificity. An attempt to improve an older commercial ELISA, the Clearview TB test, by concentrating urine prior to analysis revealed that the sensitivity could be significantly improved but also revealed a corresponding decrease in specificity (35). Similarly, the need to reduce false-positive results from the Alere LF-LAM test led the manufacturer to revise the reference card of the test toward a higher assay cutoff in 2014, which increased the specificity but decreased the sensitivity. Furthermore, the cross-reactivity of the Alere LF-LAM test to mouth-residing Actinomyces and Nocardia is likely the reason that the assay is not specific enough for LAM detection in sputum (40). We also found that both the ECL assay using the FIND 28/A194-01 pair and the Alere LF-LAM test were susceptible to cross-reactivity from environmental sources of LAM.

In addition to antibodies such as FIND 28 and CS-35 that target relatively nonspecific LAM epitopes, we also evaluated capture antibodies targeting more TB-specific structures, such as the Man2 and Man3 motifs (G3) and the MTX-Man2 and MTX-Man3 motifs (S4-20). Both provided strong signals for purified LAM from M. tuberculosis culture, but only S4-20 detected LAM in urine samples from TB patients, indicating that a large fraction of any Man2 or Man3 cap motifs in urinary LAM must present the mycobacterially biosynthesized MTX residue. This is an unexpected result, as previous studies have indicated that Man2-capped structures are dominant in the bacterial LAM, while the MTX substitution is rare and occurs only on the level of approximately one site per LAM molecule (30, 31, 41). These results indicate that there are significant differences in structures and immunoreactivity of the urinary LAM antigen and the native molecule released from bacteria.

The TB specificity of the MTX-Man motif was confirmed in cross-reactivity testing of the S4-20/A194-01 pair (Table 1). No cross-reactivity was observed for the most common organisms responsible for urinary tract infections, and in contrast to the assay employing FIND 28, we observed no detectable cross-reactivity for the LAM-producing nonmycobacterial actinomycetes. The S4-20 capture antibody also provided better discrimination of the TB-producing mycobacteria (M. tuberculosis and M. bovis) from most of the other mycobacteria species. In particular, the S4-20 capture antibody provided no detectable cross-reactivity with the fast-growing mycobacteria which produce LAM with little if any MTX modifications (26). In contrast, the FIND 28 capture antibody gave saturating or near-saturating assay signals for the tested concentrations of M. fortuitum and M. smegmatis. We also found that the S4-20/A194-01 pair was not susceptible to an unknown LAM-like contaminant introduced from our plate washers that generated false-positive signals both for the assay with the FIND 28 capture antibody and with the Alere LF-LAM test, suggesting that use of the optimal pair also prevents assay interference from environmental contaminants. We note that the TB specificity of the S4-20 antibody is also employed in an ELISA developed by Otsuka for LAM detection in sputum, which, in contrast to the Alere LF-LAM test, does not cross-react with LAM produced by prevalent oral actinomycetes species (11).

Our testing showed that pairing S4-20 with the A194-01 detection antibody provided similar detection limits as the FIND 28/A194-01 pair, but that the S4-20 capture antibody achieved significantly higher clinical specificity and provided the best overall clinical sensitivity (93%) and specificity (97%). The high overall sensitivity largely reflects the excellent sensitivity of this pair for detecting LAM in urine samples from TB-positive HIV-negative subjects (80%). Figure 3d shows a plot of the sensitivity and specificity information for the different antibody pairs (from Table 3) and also compares the observed performance to the WHO accuracy targets for POC TB tests. The results provide encouragement that the optimized assay might meet the target performances for POC TB tests for use in triage to identify patients for follow-up TB testing, as well as the more stringent requirements for use in diagnosis (42).

Although LAM was detectable in nearly all HIV-positive and the great majority of HIV-negative subjects by the S4-20/A194-01 antibody pair, our study confirmed earlier findings of increased LAM concentrations in HIV-positive subjects with low CD4 counts. Samples from TB-positive HIV-positive subjects with low CD4 counts (≤100 cells/μl) had significantly higher LAM concentrations, with selected samples having >10 ng/ml LAM. Concentrations in samples from TB-positive HIV-positive subjects with high CD4 counts (>100 cells/μl) and TB-positive immunocompetent (HIV negative) subjects were in the 11 to 1,000 pg/ml range and lower (Fig. 3b). This effect is well known from large cohort studies with the Alere LF-LAM (43). The underlying mechanisms leading to LAM antigenuria in immunocompetent and HIV-negative patients of this study remains unclear. Renal TB infection has been proposed as an explanation for high LAM concentrations in TB-HIV coinfected patients with low CD4 counts (18, 19). There is other evidence, however, suggesting that LAM is actively secreted from infected alveolar macrophages (44), supporting the lungs as a source of LAM. The active secretion of LAM would be consistent with the reported immunomodulatory properties of LAM that are likely to favor the survival of TB *in vivo* (45). The different efficacies of different capture antibodies also suggest that antigenic fragments or variants of native LAM may be secreted into the bloodstream and accumulate in the urine through glomerular filtration. A study of LAM levels in serum and their correlation with urinary levels is currently in progress.

Our study has several limitations. (i) We only used two selected samples from TB patients with low CD4 counts for the initial pairwise antibody screening, which might have biased our antibody selection: other TB patients might present different distributions of LAM structures. (ii) The reported LAM concentrations and LODs may be difficult to compare across studies due to the nonhomogeneity and variability of purified LAM standards (and the lack of an international standard), as well as differences in the ability of different antibody pairs to recognize urinary LAM relative to purified LAM standards prepared from culture. (iii) The bacterial preparations used in the cross-reactivity studies were lyophilized or frozen stocks with unknown cell concentrations, which permitted a relative comparison of the cross-reactivity of different antibody pairs but only a rough assessment of absolute cross-reactivity; a more quantitative assessment of cross-reactivity should be carried out in the future with freshly grown and quantified cell cultures. (iv) This study used a case-control design and was limited to smear-positive subjects. While this case selection should not significantly bias the direct head-to-head comparison with the Alere LF-LAM, the absolute values for diagnostic sensitivity and specificity should be treated with caution and need to be established in sufficiently powered cohort studies and in a blinded manner with predefined assay cutoffs to show the true potential of the assay. The cohort studies should be conducted in populations where the test would be used clinically.

## Supplementary Material

Supplemental file 1
