# Supplementary material for: A Novel Sensitive Immunoassay Targeting the 5-Methylthio-d-Xylofuranose–Lipoarabinomannan Epitope Meets the WHO's Performance Target for Tuberculosis Diagnosis
Source: J Clin Microbiol. 2018 Nov 27;56(12):e01338-18. doi: 10.1128/JCM.01338-18 (PMC6258851; doi:10.1128/JCM.01338-18)
Supplement: Supplemental file 1 [file zjm012186165s1.pdf]

A novel sensitive immunoassay targeting the MTX-Lipoarabinomannan epitope meets the WHO's performance target for Tuberculosis diagnosis

SUPPLEMENTARY MATERIALS

Figure S1.Oligosaccharide structures used for antibody epitope mapping.

Figure S2. Results of epitope mapping using glycan arrays.

Figure S3. Calibration curves for LAM assays.

Figure S4. Effect of heat treating urine samples on measured levels of LAM.

Figure S5. Measured assay signals for samples from clinical study.

Table S1. Analytical performance of LAM assays.

Table S2. Effect of urine matrix on LAM assay quantitation.

Supplementary Figure S1

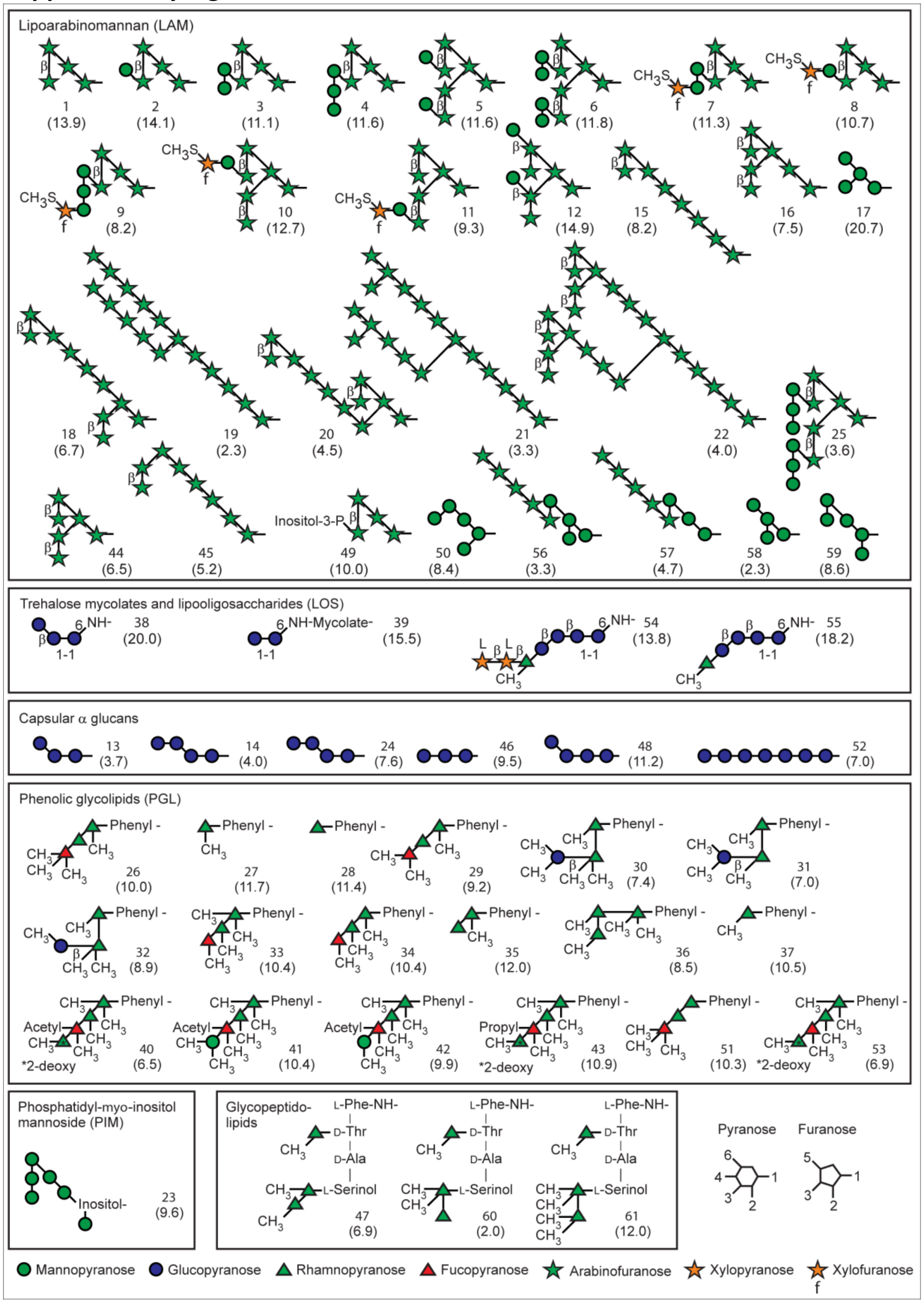

Supplementary Fig. S1. Oligosaccharide structures used for antibody epitope mapping. A total of 61 structures were used in testing. Selected oligosaccharides (Gly 16, Gly 22, and Gly 44) were further used for the development of rabbit monoclonal antibodies

Supplementary Figure S2

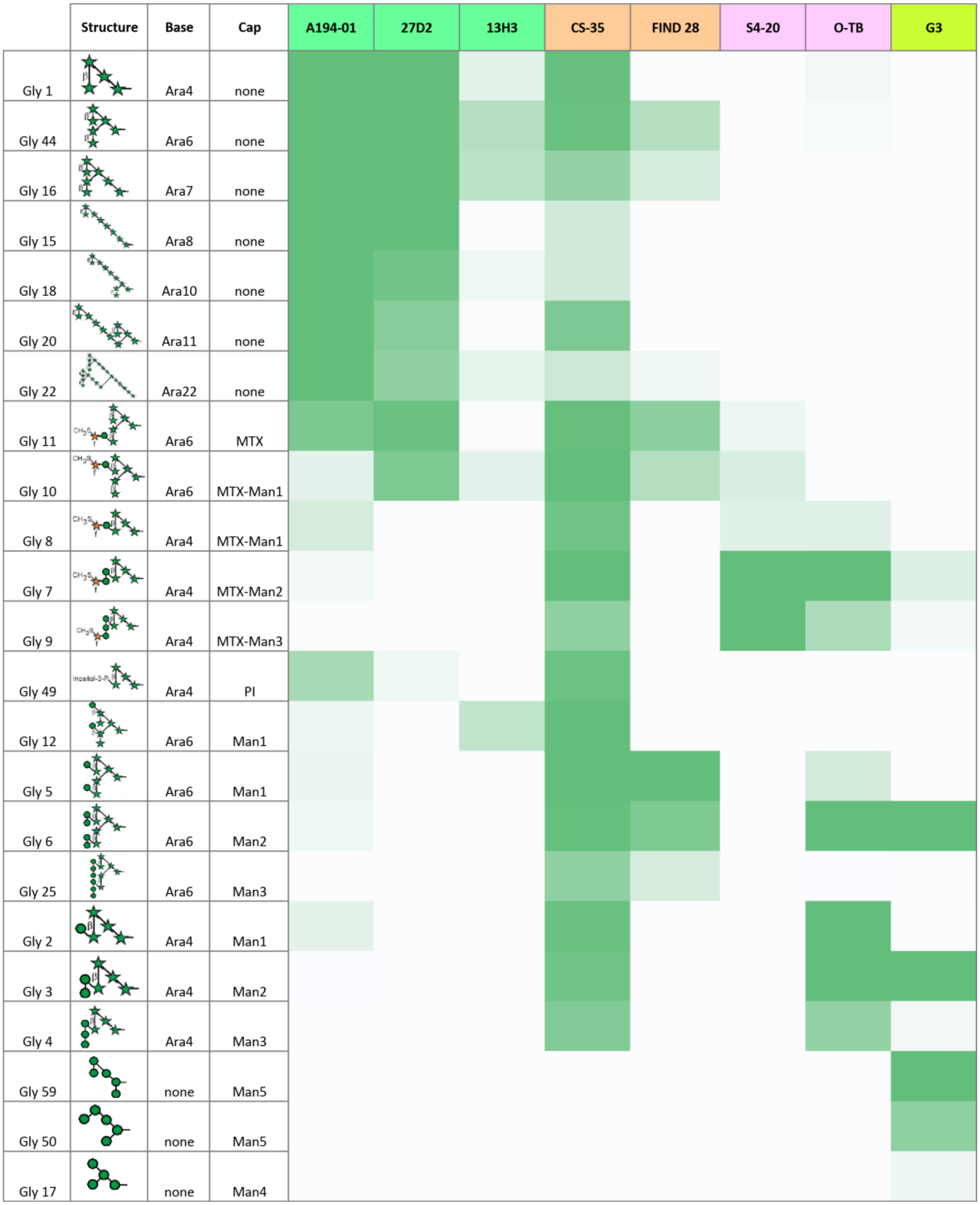

Supplementary Fig. S2. Results of epitope mapping using glycan arrays. Reactivity of monoclonal antibodies at a concentration of 0.039 µg/mL to selected oligosaccharide structures. Dark green areas represent strong binding, white areas no- or low blinding. The figure includes all glycans to which reactivity over background was shown for at least one antibody. Refer to Supplementary Figure S1 for the names used in column one.

Supplementary Figure S3

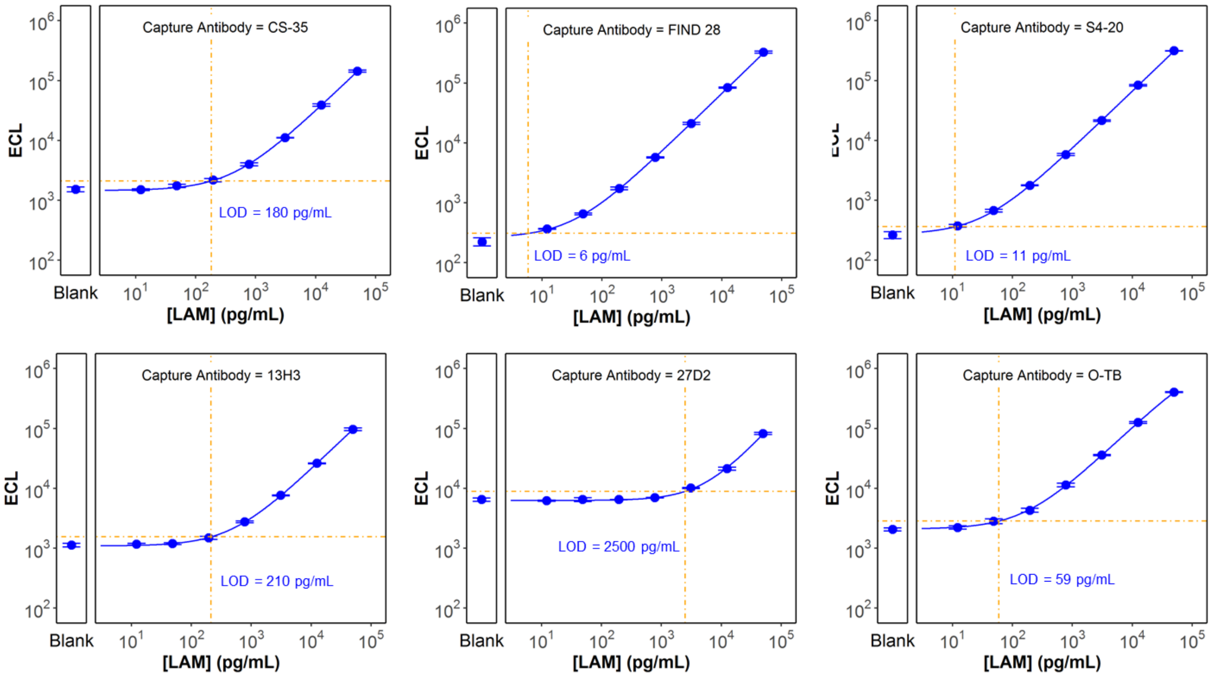

Supplementary Fig. S3. Calibration curves for LAM assays. ECL signals are plotted as a function of LAM concentration for the 6-plex panel of anti-LAM capture antibodies when multiplexed together and used in combination with the A194-01 detection antibody. The points show the measured ECL signal on each capture antibody spot when testing blank samples (n = 10) and 7 levels of LAM (n=4 per level). The x-axis is broken to show the blank sample ([LAM] = 0) on the log scale axis. The blue line shows the 4PL fit to the data. The dashed orange lines show the signal that gives an S/B ratio of 1.375 and the associated LAM limit of detection (LOD) calculated from the fitted curve.

Supplementary Figure S4

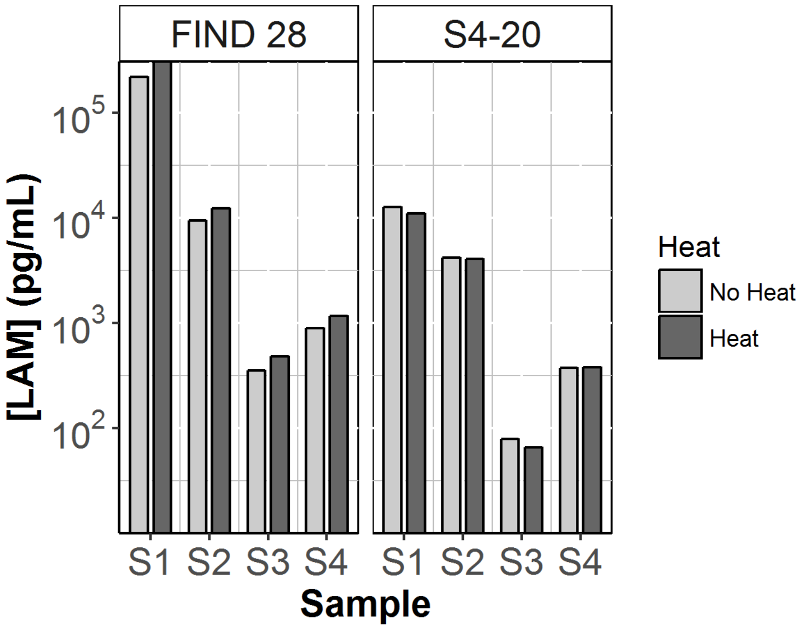

Supplementary Fig. S4. Effect of heat treating urine samples on measured levels of LAM. The plot shows the measured concentration of LAM for a set of four urine samples from TB-positive/HIV-positive patients using the FIND 28 (left panel) and S4-20 (right panel) capture antibodies. The light bars were the measured concentrations when samples were tested without pretreatment. The dark bars represent results when the samples were heat treated (85°C for 10 minutes) prior to testing.

Supplementary Figure S5

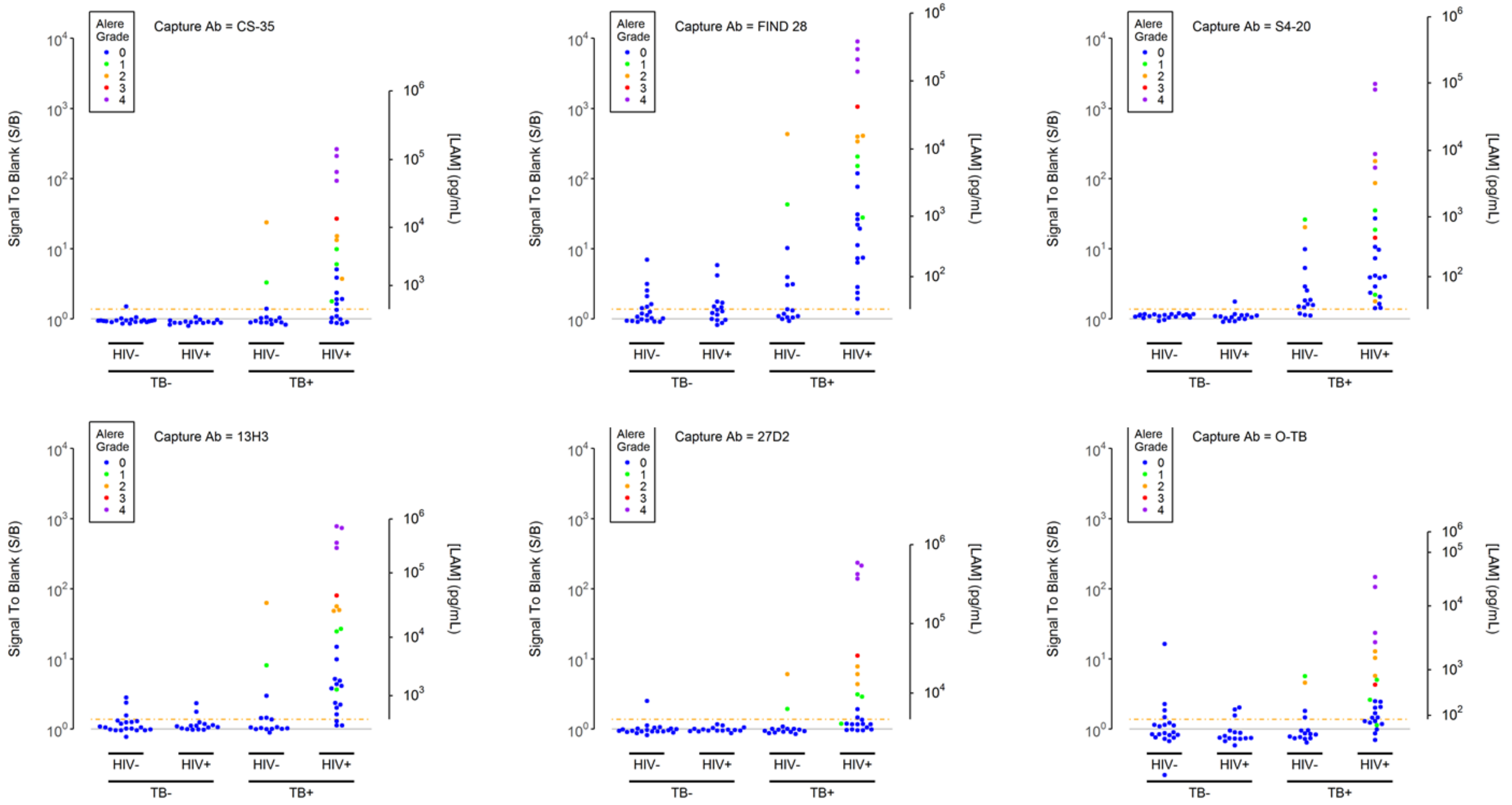

Supplementary Fig. S5. Measured assay signals for samples from clinical study. The plots show the measured signal to blank (S/B) ratios (left axis) and LAM concentrations (right axis) for each urine sample as a function of the TB and HIV status of the donor. Each plot shows the results for one of the 6 capture antibodies in the capture antibody panel when paired with the A194-01 detection antibody. The dashed orange line shows the assay threshold (S/B = 1.375). Concentration values are only meaningful for points above the assay threshold. The points are colored by the results of the Alere LF-LAM test for the same samples.

Supplementary Table S1. Analytical performance of LAM assays. The table summarizes the analytical performance of six different anti-LAM capture antibodies combined with the A194-01 detection antibody. The results were determined from eight point calibration curves as described in Figure 2. The first two data columns show average signal and CV for the blank sample (n = 14). The third shows the average CV for calibration standards providing signals above our detection threshold of 1.375-fold above the blank signal (based on four replicates per LAM concentration). The final column shows the estimates for limit of detection (LOD) based on the LAM concentration expected to give a signal equal to the detection threshold as calculated by back-fitting to the best 4-PL fit to the calibration curve.

Results Using A194-01 As Detection Antibody				
**Capture Ab**	**Blank Signal**		**Cal Signal**	**Est. LOD [LAM] (pg/mL) at S/B = 1.375**
	**ECL**	**ECL CV**	**ECL CV**	
CS-35	1,533	9%	5%	180
FIND 28	224	15%	3%	6
13H3	1,128	7%	3%	210
27D2	6,492	6%	4%	2,500
S4-20	264	12%	3%	11
O-TB	2,062	6%	5%	59

Supplementary Table S2. Effect of urine matrix on LAM assay quantitation. Matrix effects were assessed by carrying out spike recovery and dilution linearity experiments for each of the 6 capture antibodies when paired with the A194-01 detection antibody. Spike recovery is the measured LAM value for bacterial LAM spiked into a urine sample, relative to the theoretical expected value. Each entry is the average recovery for three concentrations (300, 3,000 and 30,000 pg/mL) of LAM spiked into a sample. The recovery on dilution is the measured LAM value for a diluted LAM-positive urine sample relative to the expected value based on the measured urinary LAM in the undiluted sample (as quantitated using the FIND 28 capture, the LAM levels in the undiluted samples ranged from roughly 1,000 to roughly 200,000 pg/mL). Each value is the average recovery for four dilutions ranging from 1:2 to 1:16. The table also shows the average values across all the tested samples.

		**Capture Antibody**					
		**CS-35**	**FIND 28**	**13H3**	**27D2**	**S4-20**	**O-TB**
**Sample**	**Status**	**Spike Recovery**					
1	TB+HIV+	90%	104%	100%	52%	102%	90%
2	TB+HIV+	87%	99%	106%	56%	102%	95%
3	TB+HIV+	91%	91%	98%	67%	107%	112%
4	TB-HIV-	100%	82%	95%	72%	104%	97%
5	TB-HIV-	61%	50%	101%	62%	52%	31%
6	TB-HIV-	77%	68%	90%	58%	91%	73%
7	TB-HIV-	99%	96%	100%	71%	117%	104%
**Average**		**87%**	**84%**	**99%**	**63%**	**96%**	**86%**
**Sample**	**Status**	**Recovery on Dilution**					
1	TB+HIV+	99%	108%	107%	104%	105%	126%
2	TB+HIV+	110%	144%	120%	98%	94%	157%
3	TB+HIV+	95%	135%	94%	< LOD	121%	100%
4	TB+HIV+	83%	115%	88%	< LOD	99%	110%
5	TB+HIV-	94%	104%	102%	101%	101%	125%
**Average**		**96%**	**121%**	**102%**	**101%**	**104%**	**124%**
